# Supplementary material for: Polygenic risk scores for cardiovascular diseases and type 2 diabetes
Source: PLoS One. 2022 Dec 2;17(12):e0278764. doi: 10.1371/journal.pone.0278764 (PMC9718402; doi:10.1371/journal.pone.0278764)
Supplement: S1 Table — (DOCX) [file pone.0278764.s001.docx]

S1 Table. Number, age quintile and sex of the cases and controls in the 70% training matched dataset for each of the diseases studied

| **Disease** | **Age quintile (range in years)** | **Cases** | | **Controls** | |
| --- | --- | --- | --- | --- | --- |
|  |  | **Male, N (%)** | **Female, N (%)** | **Male, N (%)** | **Female, N (%)** |
| Coronary artery disease | 1 [40.3–54.6] | 915 (72%) | 350 (28%) | 4,541 (71%) | 1,828 (29%) |
|  | 2 (54.6–60.1] | 904 (68%) | 416 (32%) | 4,354 (68%) | 2,045 (32%) |
|  | 3 (60.1–63.1] | 821 (65%) | 433 (35%) | 4,188 (66%) | 2,195 (34%) |
|  | 4 (63.1–65.9] | 834 (66%) | 434 (34%) | 4,160 (66%) | 2,186 (34%) |
|  | 5 (65.9–69.9] | 843 (67%) | 420 (33%) | 4,132 (65%) | 2,218 (35%) |
| Hypertension | 1 [40.2–53.2] | 1,096 (53%) | 960 (47%) | 4,361 (53%) | 3,793 (47%) |
|  | 2 (53.2–59.1] | 1,084 (53%) | 951 (47%) | 4,381 (53%) | 3,888 (47%) |
|  | 3 (59.1–62.6] | 1,068 (52%) | 1,000 (48%) | 4,254 (52%) | 3,920 (48%) |
|  | 4 (62.6–65.6] | 1,035 (50%) | 1,016 (50%) | 4,172 (51%) | 4,051 (49%) |
|  | 5 (65.6–69.9] | 1,020 (50%) | 1,025 (50%) | 4,043 (49%) | 4,155 (51%) |
| Atrial fibrillation | 1 [40.2–57.3] | 630 (66%) | 326 (34%) | 3,134 (65%) | 1,651 (35%) |
|  | 2 (57.3–61.5] | 606 (64%) | 343 (36%) | 3,017 (64%) | 1,723 (36%) |
|  | 3 (61.5–64.2] | 601 (62%) | 373 (38%) | 2,976 (62%) | 1,807 (38%) |
|  | 4 (64.2–66.5] | 601 (62%) | 362 (38%) | 2,996 (62%) | 1,821 (38%) |
|  | 5 (66.5–69.9] | 568 (60%) | 378 (40%) | 2,933 (61%) | 1,879 (39%) |
| Stroke | 1 [40.4–55.1] | 248 (55%) | 202 (45%) | 1,170 (55%) | 949 (45%) |
|  | 2 (55.1–60.7] | 239 (58%) | 173 (42%) | 1,220 (58%) | 892 (42%) |
|  | 3 (60.7–63.9] | 230 (56%) | 178 (44%) | 1,158 (56%) | 910 (44%) |
|  | 4 (63.9–66.5] | 232 (55%) | 189 (45%) | 1,181 (55%) | 950 (45%) |
|  | 5 (66.5–69.9] | 239 (56%) | 186 (44%) | 1,212 (56%) | 935 (44%) |
| Type 2 diabetes | 1 [40.2–52.2] | 231 (56%) | 184 (44%) | 1,189 (55%) | 958 (45%) |
|  | 2 (52.2–58.2] | 221 (53%) | 198 (47%) | 1,190 (57%) | 911 (43%) |
|  | 3 (58.2–62.0] | 231 (55%) | 192 (45%) | 1,135 (54%) | 956 (46%) |
|  | 4 (62.0–65.2] | 233 (52%) | 213 (48%) | 1,159 (53%) | 1,014 (47%) |
|  | 5 (65.2–69.9] | 246 (58%) | 179 (42%) | 1,170 (55%) | 958 (45%) |
