# Supplementary material for: Polygenic risk scores for cardiovascular diseases and type 2 diabetes
Source: PLoS One. 2022 Dec 2;17(12):e0278764. doi: 10.1371/journal.pone.0278764 (PMC9718402; doi:10.1371/journal.pone.0278764)
Supplement: Attachment — Submitted filename: Response to Reviewers.docx [file pone.0278764.s002.docx]

# Response to reviewers

**Journal Requirements:**

1. Please ensure that your manuscript meets PLOS ONE's style requirements, including those for file naming. The PLOS ONE style templates can be found at

<https://journals.plos.org/plosone/s/file?id=wjVg/PLOSOne_formatting_sample_main_body.pdf> and

<https://journals.plos.org/plosone/s/file?id=ba62/PLOSOne_formatting_sample_title_authors_affiliations.pdf>

We have ensured that our manuscript meets PLOS ONE’s style and file naming requirements.

2. Thank you for providing the following Funding Statement:

“I have read the journal's policy and the authors of this manuscript have the following competing interests: CKW, GSD, LW, NMM and RA are employees of Genetic Technologies Limited. Aspects of this manuscript are covered by Provisional Patent Application AU 2020903793, Methods of assessing risk of developing a disease. Chi Kuen Wong, Gillian Dite, Nicholas Murphy and Richard Allman are named inventors on the patent application, which is assigned to Genetic Technologies Limited.”

We note that one or more of the authors is affiliated with the funding organization, indicating the funder may have had some role in the design, data collection, analysis or preparation of your manuscript for publication; in other words, the funder played an indirect role through the participation of the co-authors.

If the funding organization did not play a role in the study design, data collection and analysis, decision to publish, or preparation of the manuscript and only provided financial support in the form of authors' salaries and/or research materials, please review your statements relating to the author contributions, and ensure you have specifically and accurately indicated the role(s) that these authors had in your study in the Author Contributions section of the online submission form. Please make any necessary amendments directly within this section of the online submission form.  Please also update your Funding Statement to include the following statement: “The funder provided support in the form of salaries for authors [insert relevant initials], but did not have any additional role in the study design, data collection and analysis, decision to publish, or preparation of the manuscript. The specific roles of these authors are articulated in the ‘author contributions’ section.”

If the funding organization did have an additional role, please state and explain that role within your Funding Statement.

We have updated the Funding Statement as follows:

The authors received no external funding for this work. CKW, GSD, LW, NMM and RA are employed by a commercial company, Genetic Technologies Limited, which provided support in the form of salaries but did not have any role in the study design, data collection and analysis, decision to publish, or preparation of the manuscript. The specific roles of all authors are articulated in the Author Contributions section.

Please also provide an updated Competing Interests Statement declaring this commercial affiliation along with any other relevant declarations relating to employment, consultancy, patents, products in development, or marketed products, etc.

Within your Competing Interests Statement, please confirm that this commercial affiliation does not alter your adherence to all PLOS ONE policies on sharing data and materials by including the following statement: "This does not alter our adherence to PLOS ONE policies on sharing data and materials.” (as detailed online in our guide for authors <http://journals.plos.org/plosone/s/competing-interests>). If this adherence statement is not accurate and there are restrictions on sharing of data and/or materials, please state these. Please note that we cannot proceed with consideration of your article until this information has been declared.

We have updated the Competing Interests Statement as follows:

I have read the journal’s policy and the authors of this manuscript have the following competing interests: CKW, GSD, LW, NMM and RA are employees of Genetic Technologies Limited. Aspects of this manuscript are covered by Provisional Patent Application AU 2020903793, Methods of assessing risk of developing a disease. CKW, GSD, NMM and RA are named inventors on the patent application, which is assigned to Genetic Technologies Limited. This does not alter our adherence to PLOS ONE policies on sharing data and materials.

**Reviewers' comments:**

**Reviewer's Responses to Questions**

1. Is the manuscript technically sound, and do the data support the conclusions?

The manuscript must describe a technically sound piece of scientific research with data that supports the conclusions. Experiments must have been conducted rigorously, with appropriate controls, replication, and sample sizes. The conclusions must be drawn appropriately based on the data presented.

Reviewer #1: Yes

Reviewer #2: Partly

2. Has the statistical analysis been performed appropriately and rigorously?

Reviewer #1: Yes

Reviewer #2: Yes

3. Have the authors made all data underlying the findings in their manuscript fully available?

The [PLOS Data policy](http://www.plosone.org/static/policies.action#sharing) requires authors to make all data underlying the findings described in their manuscript fully available without restriction, with rare exception (please refer to the Data Availability Statement in the manuscript PDF file). The data should be provided as part of the manuscript or its supporting information, or deposited to a public repository. For example, in addition to summary statistics, the data points behind means, medians and variance measures should be available. If there are restrictions on publicly sharing data—e.g. participant privacy or use of data from a third party—those must be specified.

Reviewer #1: No

Reviewer #2: No

We have now deposited the successful PRSs in PRS Catalog. These have been embargoed until publication of our paper. We have updated our Data Availability Statement as follows:

Access to the data used in this study can be obtained by applying directly to the UK Biobank at https://www.ukbiobank.ac.uk/register-apply/. The authors did not receive special access privileges to the data that others would not have. Interested researchers will be able to access the data in the same manner by applying directly to the UK Biobank.

The successful PRSs arising from this study have been deposited in PGS Catalog (PGS002773 – PGS002780).

R code used in the analyses is available from the corresponding author for non-commercial purposes only.

4. Is the manuscript presented in an intelligible fashion and written in standard English?

PLOS ONE does not copyedit accepted manuscripts, so the language in submitted articles must be clear, correct, and unambiguous. Any typographical or grammatical errors should be corrected at revision, so please note any specific errors here.

Reviewer #1: Yes

Reviewer #2: Yes

5. Review Comments to the Author

Note that we have re-numbered the reviewers’ comments for ease of cross-referencing and we are referring the new line numbers in the following responses.

Reviewer #1:

In this study, Wong et al. derive polygenic risk scores (PRS) for incident cases of several cardiovascular diseases and type 2 diabetes using a clumping and thresholding approach. They use a subset (70%) of the UKBB that they divide into age and sex matched cases and controls as a trainining set to optimize hyperparameters for their clumping and thresholding approach for each phenotype respectively. They then test the predictive performance of their PRS on the remaining 30% test set and state, that their sampling strategy controlled for the effects of age and sex in the development of the PRS.

This study has several major issues in the design of the experiment, as well as in the reporting. I think the authors need to address all of these concerns to show that their results reflect the claims they are making. I have listed alls of my comments regarding the manuscript below:

General major points

5.1: The authors mix a couple of different arguments in their abstract and introduction about how age and sex are problematic in the construction of PRS and how this apparently affects AUC reporting of predictive models. I agree with the authors, that predictive performance of PRS is often reported including age and sex as covariates in the logistic regression models and that authors should report base models without PRS as well, to properly assess how much the PRS adds ot the performance. However, the argument that age and sex are included in the PRS models themselves is questionable. The authors should clarify why they think that matching cases and controls for age and sex in the clumping and thresholding step of the PRS construction accounts for age and sex differences for traits, since the clumping and thresholding approach will select candidate variants from pre-calculated GWAS results based p-value thresholds and LD-structure in the training set. If the authors believe that age and sex are associated with the traits that they are investigating, shouldn’t they rather perform GWAS stratified by age and sex to recalibrate effect sizes and association statistics? This way, the variants that will be selected for each respective strata during clumping and thresholding would actually reflect the differences the authors want to highlight. An example of sex stratified PRS generation can be found in PMID: 35873490 (<https://pubmed.ncbi.nlm.nih.gov/35873490/>).

We have clarified that age and sex are included in the models used to report AUCs (please see our response to 5.2 below).

Ideally, we would have had summary statistics from GWAS that matched for age and sex for each of the diseases we studied. These were not available and we had to use the best available summary statistics (please see our response to 5.3 below). We could have set aside a subset of the data from the UK Biobank to calculate age- and sex-matched summary statistics but we opted not to do this so that we could maximise the data available for analysis (which was already going to be divided into testing and training sets). Although the weights from the summary statistics are used in the maxCT PRSs, we are still selecting SNPs according to the training data, which is age- and sex-matched. For the SCT PRSs, the final weights of the SNPs are obtained by fitting a penalized regression using the training data.

We have added the following to the discussion (line 267):

One potential limitation of our study is that we used summary statistics from GWAS that were not matched for age and sex. This approach will potentially reduce the performance of the PRSs. We used external summary statistics rather than obtaining them using a hold-out set from the UK Biobank so that we could maximise the samples available for analysis. While the weights from the summary statistics are used in the maxCT PRSs, we selected SNPs using the training data, which is age- and sex- matched. For the SCT PRSs, the final weights of the SNPs are obtained by fitting a penalized regression using the training data.

5.2: Related to the issue of how the authors attempt to control for age and sex in the construction of their PRS, they fail to show, that by not controlling for these covariates, models are actually over-or underperforming. The authors should generate PRS using their same approach without matching cases-and controls and report the predictive performance of these models to show whether they actually differ across sex and age groups compared to their age-and sex matched PRS.

Demonstrating the difference in performance for PRS developed by controlling for age and sex and PRS developed when not controlling for age and sex was not the aim of this paper. We simply wanted to point out the problem with reporting AUCs adjusted for age and sex without also reporting AUCs for PRS alone.

A strength of our study is that the discriminatory performances of our PRSs are solely due to genetic factors. We do not want the reader to compare our AUCs for PRS alone with AUCs for PRS and other covariates on other studies.

Careful attention to age and sex is essential in developing a PRS because these can confound the association between the disease of interest and the PRS. While other studies adjust for age and sex by including them in their model, they often don’t provide enough information to be able to assess the independent contribution of the PRS to risk prediction. An AUC derived from a model with a PRS, age and sex tells us nothing of the contribution of the genetic component. To provide an example of this we did one comparison in lines 241–244. Using age and sex as the base model, adding the PRS improves the AUC by 0.027, while using the PRS as the base model, adding age and sex improves the AUC by 0.125. To assess the contribution of the PRS, the AUC needs to be reported for the PRS alone.

We have re-written the beginning of paragraph 3 of the introduction to clarify the problems with AUCs reported in other studies:

However, the prediction models used in these studies adjust for age and sex, which are known risk factors for the disease of interest and confound the association between the PRS and the disease. The reference models in these studies, which often include age, sex and a few principal components, already have high AUCs. If these studies do not report AUCs separately for the reference model and the PRS, recognizing how much a PRS actually contributes to disease prediction is impossible.

We have also made some changes to the discussion. The first paragraph is now:

In this study, we have addressed two important limitations of some other studies that have attempted to develop PRSs [1,2,5]. First, we have ensured that our PRS models do not include the effects of age and sex and represent the genetic effects alone.

The second part of paragraph 2 (from line 240) is now:

Importantly, the AUCs for our PRSs are due solely to genetic effects and are not inflated from the inclusion of age and sex in the model. For example, in the testing dataset for atrial fibrillation, the AUC for a base model with age and sex was 0.711, while the AUC for our PRS alone was 0.613. If we present the AUC for PRS, age and sex – as other authors [1,5] have done – it would be 0.738. This age- and sex-adjusted AUC has often been reported without also reporting an AUC for the PRS alone, making it difficult to understand the contribution of the PRS to disease prediction.

5.3: The authors removed prevalent disease cases from their analysis. They do highlight that this is to prevent biased estimation of disease risk, which is an important point to make. However, they fail to also highlight that by using prevalent disease based GWAS summary statistics in the selection of their PRS variants, their PRS might also be underperforming, as variants selected for PRS might be biased towards prevalent disease.

We would have preferred to use summary statistics for GWAS that used only incident cases but unfortunately these were not available in the GWAS Catalog that we used. We needed to select summary statistics that were not generated using the UK Biobank and that were obtained from Caucasian populations.

We have added text beginning at line 220 to explain why we chose the summary statistics for each disease.

For each disease, we selected summary statistics from the GWAS Catalog that are publicly available for download, discovered using mostly Caucasian populations with a large sample size, and not generated using the UK Biobank. Ideally, we would prefer to use summary statistics that used only incident cases (and also satisfied other mentioned criteria) in order to match our study design but we cannot find such summary statistics. We would expect better performance if the summary statistics were commensurate with our study design. Note that for hypertension, the summary statistics were generated using the UK Biobank so the performance of the hypertension PRS could be overestimated.

5.4: In the methods section, the authors write that they computed AUC values to assess predictive performance of their PRS, but fail to provide details about which computational packages they used (if they used any) to do this. In the results section, they refer to using logistic regression to calculate the Odds ratio (OR) per standard deviation for Table 3. Were the probabilities from the logistic regression models used for calculation of AUC and did the authors include any covariates in the logistic regression models (PCs, smoking, statin usage, etc?). The authors need to add the details about logistic regression models for OR per SD calculation to the methods section. Currently, what they write in the results section does not match what they report in the methods section for OR per SD calculation.

The AUCs were calculated separately from the logistic regression to calculate the odds ratio per standard deviation. The AUCs were calculated using the bigstatsr package in R AUC and no covariates were included in the model.

We have changed the final paragraph of the methods as follows:

After PRSs were created for each disease, we quantified their predictive power in the testing data by using the bigstatsr package in R to compute AUCs. No other covariates were included in the calculation of the AUCs. To assess the association of each of the PRS with the disease of interest in the testing data, we used logistic regression to estimate the odds ratio (OR) per standard deviation (SD) of the PRS. The SDs were calculated using the controls in the 30% testing dataset.

Minor points

5.5: One controversial point the authors raise is whether variant imputation is too computationally and analytically intense to be generally applied to PRS calculation. They do cite a study that investigated the effect of different imputation algorithms on PRS performance (Ref 8 in the manuscript : <https://genomemedicine.biomedcentral.com/articles/10.1186/s13073-020-00801-x>). However, while this study found that imputation can introduce variability on the individual level, it generally does not cause problems in interpretation of the PRS. As many easy-to-use public tools such as the Michigan Imputation server (<https://imputationserver.sph.umich.edu/index.html>) are providing low-cost, fast imputation to large reference panels and whole-genome sequencing is becoming cheaper and cheaper, the argument that imputation and large PRS panels are unfeasible becomes less valid. The Michigan imputation server has recently even incorporated PRS calculation (from PGS catalog) as part of the imputation process, making it even easier and faster to obtain PRS for datasets.

As Reviewer 2 has pointed out, discussion of the use of genotyped vs imputed data is well beyond the scope of this paper. We have removed this discussion from the paper and will address this issue in future work. Please see response to 5.10 for more details.

Data reporting

5.6: The authors fail to mention availability of their score for the public to reproduce their results in independent cohorts. They don’t provide any reference to a data or any other repository where their PRS variants and effect sizes can be found. I suggest the authors submit their PRS to <https://www.pgscatalog.org/>, a resource that has been created for exactly the purpose of making PRS reporting more reproducible.

We have updated our Data Availability Statement and have uploaded the PRSs to PGS Catalog. See Question 3 above for full details.

Reviewer #2:

The authors describe that AUC reflects the predictive accuracy of not only the polygenic score but also important risk factors, namely age and sex. The authors use sex- and age-matched control samples to estimate the AUC of polygenic score alone.

I have some major concerns. Firstly, it is not very clear to me what the aim of this work is and what contribution to the field the authors are trying make.

5.7: If the aim is to show that the commonly used AUC estimates are ‘inflated’ due to the effects of age and sex, what is missing in the manuscript is a proper comparison between AUC estimates using the same polygenic scores in case control samples with and without matching the distributions of sex and age. It was only briefly mentioned in the discussion section (line 139). It would always be helpful to quantify the observations.

Please see the response to 5.2.

5.8: If the aim is to develop polygenic scores that improve risk stratification for the tested traits (as mentioned on line 65 in the introduction), the authors did not compare their PRS with previously published scores, some of which used more sophisticated methods such as Bayesian based methods (e.g. LDpred, PRSCS, and SBayesR). These methods are believed to outperform C+T based methods. It can probably also address the issue that the stroke score was not predictive, by using the PRS developed by Neumann et al. (available at the PGS Catalog; <https://www.ahajournals.org/doi/10.1161/STROKEAHA.120.033670#d6462236e293)or> from an older study that the authors themselves cited (reference 3).

Comparing our PRSs to those developed by others was beyond the scope of this paper and will be undertaken for a future paper.

We have removed the phrase “that improve risk stratification” from line 68.

5.9: Moreover, I don’t agree with the authors that adjusting or considering age and sex in the model when reporting the ‘inflated’ AUC of a polygenic score is an issue. We compare AUC basically in the following two scenarios. (1) We compare different polygenic scores or scores generated using different parameters in the same testing sample. Age, sex and other baseline characteristics have the same effects on the goodness-of-fit metrics and better scores always have higher AUC. (2) We compare scores that are validated in different cohorts. It is more complicated and there are always other cohort-specific factors (such as different healthcare systems, phenotype definitions, fine-scale population stratification, different socioeconomic status) which may contribute to the estimation of PRS accuracy. Even the case control samples within each cohort have matched age and sex distributions, differences in the distributions between cohorts (e.g. one cohort might have many young cases while the other cohort recruits more older cases) could still result in AUC estimates that are not comparable. Also, power is reduced when selecting controls that match with cases due to the smaller sample size. There are other approaches to accounting differences in age and sex between cases and controls, which have been commonly used already, such as the incremental R2 (AUC or pseudo-R2 for binary traits; <https://doi.org/10.1038/s41596-020-0353-1>) which quantifies the increase in variance explained with the addition of the PRS to the baseline model.

We are concerned with the uncritical comparison of AUCs as the endpoints of studies of PRSs without taking into account how those AUCs were derived (scenario 2 in the comment above).

We have re-written the paragraph beginning on line 58 as follows:

In addition, comparison of AUCs obtained by including additional covariates between studies can be difficult because of differences in the age and sex distributions of the study sample. A disease with a non-linear association with age will have a different AUC in a study of younger people versus a study of older people. This is because the reference models (i.e., the age and sex models) have different AUCs. Not knowing the separate AUCs for the PRS and the reference model makes comparison difficult.

We chose to age- and sex-match our controls, rather than adjust for age and sex, because we did not have to make any assumption about the nature of the association with age (e.g., linear or non-linear) and the presence of any interaction between age and the PRS.

5.10: Also, the authors discussed a lot about the disadvantages of using imputed versus genotyped array data, which doesn’t really fit in the manuscript. The authors did not do any analysis comparing the accuracy, individual-level variability, or costs versus benefits between PRS with a small number of genetic variants using array data and PRS with many more genetic variants using imputed data. This is out of the scope of the work and the discussion on this topic probably needs to be shortened. The justification of using array data can be briefly mentioned in the beginning of the results section when the study samples and analysis methods are introduced, or in the methods section.
More specific comments:

We agree that the discussion of genotyped vs imputed data is beyond the scope of the paper. This is of interest to us, and has been the subject of discussions in the laboratory. We will consider this issue in a separate study.

We have removed discussion of genotyped vs imputed data from the discussion.

We have clarified that we have used genotyped data in the participants section of the Material and Methods.

5.11: Results section on page 5: it would be great to briefly introduce the study cohort first. More specifically, the effort of matching controls with cases in terms of age and sex should be mentioned, as well as the sample sizes. It would be great to move table 4 here.

We have moved Table 4 to the beginning of the results (and we have re-numbered other tables accordingly) and have provided detail on the age- and sex-matching in the training dataset in Supplementary Table 1. The results section now begins as follows:

S1 Table shows, for each disease, the number of participants in the 70% age- and sex-matched training dataset and the number in the 30% unmatched testing dataset. Five controls were able to be selected for almost all cases; three controls were not able to be matched for coronary artery disease, atrial fibrillation and stroke. For hypertension, four controls were drawn for each case and two controls were not able to be matched.

S1 Table. Number, age quintile and sex of the cases and controls in the 70% training matched dataset for each of the diseases studied

| **Disease** | **Age quintile (range in years)** | **Cases** | | **Controls** | |
| --- | --- | --- | --- | --- | --- |
|  |  | **Male, N (%)** | **Female, N (%)** | **Male, N (%)** | **Female, N (%)** |
| Coronary artery disease | 1 [40.3–54.6] | 915 (72%) | 350 (28%) | 4,541 (71%) | 1,828 (29%) |
|  | 2 (54.6–60.1] | 904 (68%) | 416 (32%) | 4,354 (68%) | 2,045 (32%) |
|  | 3 (60.1–63.1] | 821 (65%) | 433 (35%) | 4,188 (66%) | 2,195 (34%) |
|  | 4 (63.1–65.9] | 834 (66%) | 434 (34%) | 4,160 (66%) | 2,186 (34%) |
|  | 5 (65.9–69.9] | 843 (67%) | 420 (33%) | 4,132 (65%) | 2,218 (35%) |
| Hypertension | 1 [40.2–53.2] | 1,096 (53%) | 960 (47%) | 4,361 (53%) | 3,793 (47%) |
|  | 2 (53.2–59.1] | 1,084 (53%) | 951 (47%) | 4,381 (53%) | 3,888 (47%) |
|  | 3 (59.1–62.6] | 1,068 (52%) | 1,000 (48%) | 4,254 (52%) | 3,920 (48%) |
|  | 4 (62.6–65.6] | 1,035 (50%) | 1,016 (50%) | 4,172 (51%) | 4,051 (49%) |
|  | 5 (65.6–69.9] | 1,020 (50%) | 1,025 (50%) | 4,043 (49%) | 4,155 (51%) |
| Atrial fibrillation | 1 [40.2–57.3] | 630 (66%) | 326 (34%) | 3,134 (65%) | 1,651 (35%) |
|  | 2 (57.3–61.5] | 606 (64%) | 343 (36%) | 3,017 (64%) | 1,723 (36%) |
|  | 3 (61.5–64.2] | 601 (62%) | 373 (38%) | 2,976 (62%) | 1,807 (38%) |
|  | 4 (64.2–66.5] | 601 (62%) | 362 (38%) | 2,996 (62%) | 1,821 (38%) |
|  | 5 (66.5–69.9] | 568 (60%) | 378 (40%) | 2,933 (61%) | 1,879 (39%) |
| Stroke | 1 [40.4–55.1] | 248 (55%) | 202 (45%) | 1,170 (55%) | 949 (45%) |
|  | 2 (55.1–60.7] | 239 (58%) | 173 (42%) | 1,220 (58%) | 892 (42%) |
|  | 3 (60.7–63.9] | 230 (56%) | 178 (44%) | 1,158 (56%) | 910 (44%) |
|  | 4 (63.9–66.5] | 232 (55%) | 189 (45%) | 1,181 (55%) | 950 (45%) |
|  | 5 (66.5–69.9] | 239 (56%) | 186 (44%) | 1,212 (56%) | 935 (44%) |
| Type 2 diabetes | 1 [40.2–52.2] | 231 (56%) | 184 (44%) | 1,189 (55%) | 958 (45%) |
|  | 2 (52.2–58.2] | 221 (53%) | 198 (47%) | 1,190 (57%) | 911 (43%) |
|  | 3 (58.2–62.0] | 231 (55%) | 192 (45%) | 1,135 (54%) | 956 (46%) |
|  | 4 (62.0–65.2] | 233 (52%) | 213 (48%) | 1,159 (53%) | 1,014 (47%) |
|  | 5 (65.2–69.9] | 246 (58%) | 179 (42%) | 1,170 (55%) | 958 (45%) |

5.12: Line 76: figure 2 shows the distributions, not figure 1.

We have updated the names of the figure files to refer to the correct figures.

5.13: Page 7: I’m not very sure whether the comparison of SCT and maxCT scores is necessary here. It has been clearly established in the original paper (citation 7) that the SCT scores show better performance than maxCT. It is not surprising either that the SCT scores contain more genetic variants given that they are combinations of multiple C+T scores.

While we are aware that SCT PRSs perform better than maxCT PRS in terms of discrimination between cases and controls, we wanted to understand by how much. We wanted to know if the predictive performances were similar for the maxCT and SCT PRSs.

Our interest stems from issues that arise in the laboratory implementation of the scores; extremely large numbers of SNPs are more difficult to implement than smaller numbers of SNPs – in terms of data storage requirements and the work that must be undertaken to validate laboratory procedures.

5.14: Line 151: it would be more accurate to say that “our results suggest that PRSs that were developed in this study have xxx”. The conclusion may not be generalisable to other PRSs.

We have updated the beginning of the sentence as suggested by the reviewer and have made a similar change to the conclusion:

Our results suggest that the PRSs that were developed in this study have…

5.15: Line 168-169: it should be made clearly in the beginning of the results section that the genotyped data were used.

We have clarified that we used genotyped data in the material and methods (line 83).

5.16: Page 10-11: like I said previously, there are a lot of discussions about imputed data, which is not the focus of this manuscript which currently does not have any analysis relevant to imputed vs genotyped data.

This has been cut from the paper (see response to 5.10).

5.17: The authors should also consider that genotyping only a small number of SNPs that are used in the maxCT scores is not flexible. There will be larger GWAS and more accurate PRS for the tested diseases as well as new phenotypes in the future, and different genetic markers will be needed. In the long term, using commercial array chips + imputation strategy is probably more cost effective, as we can reuse the high coverage data for better scores and scores for many other traits when available.

While genotyping the smaller number of SNPs used in the maxCT scores may be less flexible than a whole genome sequencing approach, current genotyping arrays comprise in excess of 650,000 SNPs and have not proved limiting in our hands, particularly when supplemented by imputation. As the reviewer suggests, the content of genotyping arrays is likely to evolve as larger GWAS refine the PRS for tested diseases. We agree with the reviewers thoughts on cost-effectiveness of an array-based genotyping approach, which was one of the contributing factors to our study design.

5.18: Line 171-172: what does the ‘validation process’ mean here?

This has been cut from the paper (see response to 5.10).

5.19: Line 185-186: please provide reference to this claim which I find hard to believe. There are standard ways to perform QC of array data and imputation can be done easily and freely using online servers.

This has been cut from the paper (see response to 5.10).
