# Supplementary material for: Polygenic risk scores for cardiovascular diseases and type 2 diabetes
Source: PLoS One. 2022 Dec 2;17(12):e0278764. doi: 10.1371/journal.pone.0278764 (PMC9718402; doi:10.1371/journal.pone.0278764)
Supplement: Attachment — Submitted filename: Response to Reviewers.docx [file pone.0278764.s003.docx]

# Response to reviewers

6. Review Comments to the Author

Reviewer #1: The authors have adequately addressed all of my comments to their original manuscript submission. The scores submitted to the PGS Catalog need to be made public once the manuscript is accepted in a publication journal.

We will ensure that the PRSs submitted to the PGS Catalog are made public when the paper is accepted for publication.

Reviewer #2: The authors have addressed most of my comments and the revised manuscript has been greatly improved. I still have one last comment on authors’ response to 5.2 and 5.7. It is important to establish and quantify the issue of over-estimation, as the authors claimed, of PRS accuracy measured in AUC when including age and sex. I still think that it would be great to analyse all traits in addition to atrial fibrillation (line 241-244), and add the “over-estimated” AUC somewhere in a main table. I find it interesting and helpful (and perhaps some other readers too) to know how much the AUC metrics reported in other studies are overestimated due to the effects of covariates such as age and sex.

In additional to atrial fibrillation, we have now added Table 7 to show the inflation of AUCs when age and sex are included for all five diseases.
